# Associations between Dental Anxiety Levels, Self-Reported Oral Health, Previous Unpleasant Dental Experiences, and Behavioural Reactions in Dental Settings: An Adult E-Survey

**DOI:** 10.3390/medicina60081303

**Published:** 2024-08-12

**Authors:** Ruzica Peric, Antonija Tadin

**Affiliations:** 1 Department of Restorative Dental Medicine and Endodontics, University of Split School of Medicine, 21000 Split, Croatia; rp91808@mefst.hr; 2 Department of Maxillofacial Surgery, Clinical Hospital Centre Split, 21000 Split, Croatia

**Keywords:** adults, dental anxiety, dental fear, Modified Dental Anxiety Scale, prevalence, oral health

## Abstract

*Background and Objectives*: The aim of this study was to investigate the prevalence of dental anxiety, its association with self-reported oral health, and sociodemographic factors in adults that are critical for improving oral health and well-being. *Materials and Methods*: An online survey was conducted via social media, with 1551 adults (76.5% women, 23.5% men) participating nationwide. Data collected included demographic data, Modified Dental Anxiety Scale (MDAS) scores, and associations between dental anxiety, negative experiences, and self-reported oral health. The analysis included psychophysiological, behavioural, and emotional responses and avoidance of dental visits, using descriptive and generalised linear regression models. *Results*: This study found that the mean score of the MDAS was 9.70 ± 5.11 out of 25, 19.1% of the participants reported no dental anxiety, and 7.8% suffered from dental phobia. Gender, age, and socioeconomic status had no significant effect on the prevalence of anxiety. Although more than half of the participants reported negative dental experiences, particularly in childhood, anxiety levels were unaffected. However, those who rated their oral health as excellent or very good had lower anxiety scores (*p* = 0.008, *p* = 0.024). Among the dental procedures, oral surgery (58.7%) and prosthetic (restorative) dental treatments (15.2%) caused the most anxiety. Avoidance behaviour correlated with increased anxiety (*p* ≤ 0.001), as did postponing dental visits until severe pain occurred (*p* = 0.011). *Conclusions*: These results emphasise the significant prevalence of dental anxiety in adults, particularly for surgical procedures and drilling, posing challenges in patient management. Tailored strategies are essential to reduce anxiety, improve patient well-being, and optimise dental service delivery and treatment efficacy.

## 1. Introduction

Dental anxiety and dental fear are still major problems widespread in both the adult and paediatric populations worldwide. Dental anxiety, dental fear, and dental phobia present significant hurdles for both patients and dentists. Although these terms are often used interchangeably, there are nuanced distinctions [1].

The prevalence of dental anxiety in the adult population is reported very variably in the literature. Figures range from 4.2% to over 50.0% and reflect considerable cultural, social, and economic differences. Recent data estimate the prevalence of dental anxiety at 15.3%, with 12.4% of adults experiencing a high level of anxiety and fear and around 3.3% experiencing a severe level of anxiety [2]. In young children, the prevalence of dental anxiety also varies widely, ranging from 4% to 98% [3].

Various factors can influence the occurrence of dental anxiety, including individual characteristics such as age and gender, previous experiences with dentists, and environmental and socioeconomic conditions [4,5,6,7]. Dental anxiety is also related to the patient’s personality such as extraversion or neuroticism and their psychological status. Studies have shown that women are more likely to suffer from dental anxiety compared to men. In addition, anxiety is generally more pronounced in younger patients than in older patients [8,9].

According to the current knowledge, people with dental anxiety often avoid or delay dental appointments, which leads to a deterioration in their oral health and consequently to a poorer quality of life. Moreover, people with high levels of anxiety and fear typically have poor oral hygiene, leading to a higher prevalence of tooth decay and tooth loss. This cycle of avoidance and deterioration can significantly impact the overall well-being of those affected and lead to more extensive dental interventions in the future [10,11]. Numerous studies around the world have investigated this phenomenon, in different age groups, from children to adults, and various measurement tools have been developed to assess the level of dental anxiety [12,13,14,15,16,17,18,19,20,21]. Some instruments focus primarily on assessing the pain felt during dental treatment, while others assess the dynamics of the patient–dentist relationship. However, most scales are designed to assess clinical situations in dental practice [22].

In the Republic of Croatia, several studies have been conducted to determine the prevalence of dental anxiety, fear, or phobia associated with various dental procedures, in the paediatric population [23,24,25,26,27], adolescents [28,29], and adults [30]. According to a 2022 study using the Corah Dental Anxiety Scale, 12.5% of children and 8.3% of parents had high levels of dental anxiety [23]. Similar results were produced in a study of children in 2024 [24], while an earlier study found high levels of dental anxiety in 29% of children [25]. Risk factors include gender, with a higher prevalence in girls than boys, previous unpleasant treatment experiences, and parental dental anxiety [23,24]. When considering the available data, it appears that there are limited data on dental-treatment-related anxiety, its prevalence, and its impact on adult oral health in Croatia.

The objectives of this study were (1) to determine the prevalence of dental anxiety in adults, (2) to assess the relationship between sociodemographic characteristics and the level of dental anxiety, (3) to examine the correlation between the level of dental anxiety and the respondents’ subjective perceptions of oral health, and (4) to determine the impact of dental anxiety on behaviours such as avoidance of dental visits. This study is unique because it is the first cross-sectional study in the Croatian adult population in which the Modified Dental Anxiety Scale (MDAS) has been used. The MDAS provides a detailed understanding of dental anxiety. In addition, this study examined the relationship between dental anxiety and sociodemographic factors, subjective perceptions of oral health, and previous dental experiences, allowing for a comprehensive analysis of its impact on adult oral health.

## 2. Materials and Methods

### 2.1. Study Design and Population

This cross-sectional E-survey was conducted in January and February 2024 at the Department of Restorative Dentistry and Endodontics, Faculty of Medicine, University of Split, with the approval of the Ethics Committee of the Faculty of Medicine (Ref. No.: 2181-198-03-04-23-0081, Class: 003-08/23-03/0015). The data were collected using an online survey tool (Google Forms, Google, Mountain View, CA, USA), with the link to the survey distributed via social media platforms (Facebook, WhatsApp). Participants were asked to share the survey link with their acquaintances to contribute to the representativeness of the sample from the general population, using the “snowballing” method. The survey began with information about the research and its aims, and completion of the questionnaire was taken as consent to participate in this study. Participation in this research was voluntary and completely anonymous. No data were collected that could be used to identify the participants. All methods were conducted in accordance with the relevant guidelines and regulations. This study was published in accordance with the Checklist for Reporting Results of Internet E-Surveys (CHERRIES) and the Strengthening the Reporting of Observational Studies in Epidemiology (STROBE) reporting checklist [31].

The questionnaire was completed by 1551 participants, including 1186 women and 365 men. The inclusion criteria were adults who were willing to complete the survey, resided in the territory of the Republic of Croatia, and were of legal age. Incomplete questionnaires, participants living outside the Republic of Croatia, and persons younger than 18 years of age were excluded from this study. The minimum sample size required to successfully conduct this study (n = 385) was calculated using the Sample Size Calculator computer program (Inc.RaoSoft^®^, Seattle, WA, USA). The calculation was based on the number of adult residents of the Republic of Croatia according to the last census (n = 3,223,679) from 2022, with a confidence interval of 95 and a margin of error of 5 [32].

### 2.2. Questionnaire

The data in this study were gathered using a questionnaire consisting of 67 questions divided into eight sections. This questionnaire was composed of questions used in similar studies and adapted to the needs of this study [8,10,22,33,34,35]. The survey was originally designed by a professor of endodontics and restorative dentistry in collaboration with a dental student. To ensure content validity, it was subsequently reviewed and revised by a professor of paediatric dentistry. The revised survey was pilot tested on a sample of 20 individuals who met the inclusion criteria. These participants evaluated the user-friendliness and technical functionality of the electronic survey. The pilot test took place online with respondents who did not take part in the primary study. Based on the feedback and results of the pilot survey, adjustments were made to improve the clarity and comprehensibility of the questionnaire items. Cronbach’s alpha coefficient for the internal consistency of the MDAS questionnaire tested on the pilot study participants was 0.784, indicating acceptable reliability. The questionnaire took approximately 10 to 15 min to complete.

The questionnaire began with five general questions (Q1–Q5) about the participants, including their gender, age, level of academic education, employment status, and socioeconomic status. The second part of the questionnaire included a measurement tool to assess the participant’s level of anxiety and dental fear. The Modified Dental Anxiety Scale (MDAS) included five questions on self-rated emotions related to an upcoming dental appointment, feelings in the dental office waiting room, the sensations of drilling and polishing the teeth, and awareness of the need for local anaesthesia (Q6–Q10). Each item was scored as follows: 1 = no anxiety; 2 = mildly anxious; 3 = moderately anxious; 4 = severely anxious; 5 = extremely anxious. The total score was the sum of the scores for all five responses, with a minimum score of 5 and a maximum score of 25. The interpretation of the scale scores for the patients was as follows: 5 = no anxiety; 6–7 = extremely low anxiety; 8–10 = low anxiety; 11–15 = moderate anxiety; 16–19 = severe anxiety; >19 = dental phobia [36]. The third section of this questionnaire consisted of seven questions that mapped the presence of dental anxiety, experiences of unpleasant dental encounters, and the first unpleasant experience (Q11–Q17). Participants had to provide information on the acquisition of anxiety and the need for painkillers, sedatives, or local anaesthesia before potentially painful dental treatments. The fourth section comprised eight questions on self-assessed oral health and its importance, the number of teeth extracted, the number of teeth with fillings or crowns, bleeding gums, bad breath, current pain in the mouth, and the frequency of visits to the dentist (Q18–Q25). The fifth section of the questionnaire consisted of nine questions with dichotomous answer options (“yes” and “no”), in which participants were asked to indicate which dental procedures caused them anxiety. The available options included endodontic, orthodontic, oral surgery, prosthetic/restorative, and periodontal procedures as well as local anaesthesia (Q26–Q34). The sixth part of the questionnaire comprised 12 multiple-choice questions on factors related to a fear of dentists (Q35–Q46). The predetermined responses included feelings of helplessness, the smell in the dental office, discomfort, clinical errors or injuries caused by the dentist, fear of procedures, consulting a doctor, reading online information about dental procedures, waiting for treatment, noisy patients in the waiting room, the cost, or none of the above. Participants could choose either “yes” or “no” for each question. The seventh section was divided into 15 questions on psychophysiological, behavioural, and emotional reactions during a dental visit (Q47–Q61). These reactions included muscle tension in the legs, black spots in the visual field, difficulty breathing, feeling hot, nausea, palpitations, dry mouth, dizziness or light headedness, abdominal discomfort, numbness or tingling, fainting, inability to relax, choking, trembling hands, and facial flushing. Participants could answer “almost never”, “sometimes”, “often”, or “always” to each occurrence. The questionnaire concluded with the last, eighth section, which contained six questions on the following topics: Avoiding dental visits for as long as possible or until severe pain occurs, missing scheduled appointments due to anxiety, cancelling appointments due to anxiety, arriving at the office but leaving before entering, and being encouraged by another person to see the dentist due to pain (Q62–Q67). Participants answered either “yes” or “no” to each of the questions asked.

### 2.3. Statistical Analysis

For data analysis, the Statistical Package for the Social Sciences, version 26 (SPSS, IBM Corp, Armonk, New York, NY, USA) was utilised. The level of statistical significance set for data examination was established at *p* < 0.05. Normality of the response distribution was assessed using the Kolmogorov–Smirnov test. Descriptive analysis was employed to calculate frequencies and percentages, while quantitative data were presented in the form of medians and interquartile ranges. A generalised linear model was constructed to examine the influences of demographic factors, the self-assessed oral health status, negative dental experiences, and behaviours in the dental office on the degree of dental anxiety. The MDAS score was divided into two categories: “low”, defined as below the median, and “high”, defined as at the median level and above. A “low” MDAS score was used as the reference value.

## 3. Results

Table 1 shows the demographic and socioeconomic characteristics of the participants in relation to the degree of dental anxiety. This study was conducted with 1551 participants, the majority of whom were female (76.5%, n = 1186). The mean age of the participants was 34.66 ± 13.83 years (Md = 34.0, IQR 24.0–47.0, Min = 18, Max = 95). According to the MDAS, participants with a bachelor’s degree (*p* = 0.013) and an MSc/PhD degree (*p* = 0.035) showed a significantly higher level of dental anxiety than participants with a lower level of education. In addition, professionals showed significantly higher levels of dental anxiety compared to students (*p* = 0.007). The MDAS showed that about one-fifth of the participants (19.1%, n = 297) belonged to the category without dental anxiety. One-quarter of the participants (25.7%, n = 399 and 26.6%, n = 413) were categorised as having extremely low anxiety, and the same was true for low anxiety. The mild anxiety category included a smaller proportion of participants, only 14.4% (n = 223). We found that 6.3% (n = 98) and 7.8% (n = 121) of the participants belonged to the severely anxious and extremely anxious groups respectively, indicating a phobia. The average score of dental anxiety according to the MDAS for all participants was 9.70 ± 5.11 (Md = 8.0; IQR 6.0–11.0; Min = 5; Max = 25). For about 55.0% of the participants, the anxiety score was at or above the median value.

Table 2 shows the distribution of participants according to their responses on the Modified Dental Anxiety Scale. Here, 12.6% of the participants stated that drilling the teeth caused them the greatest anxiety (n = 195). In contrast, an astonishing 61.2% (n = 949) of participants stated that the anticipation of a dental appointment the next day did not cause them any anxiety at all, while 54.7% (n = 844) stated that tooth cleaning and polishing did not cause them any anxiety.

Table 3 shows respondents’ answers related to the presence of dental anxiety, negative experiences at the dentist’s, and higher levels of anxiety on the MDAS. Approximately one-fifth of respondents had never experienced a negative dental event (20.9%). An astonishing 52.9% (n = 820) of respondents reported having had their first unpleasant experience in childhood, and in 36.2% of cases, this experience was personal. The vast majority, 80.1% (n = 1243), never take painkillers before treatment, while only 25.9% (n = 402) always request local anaesthesia before procedures. According to the MDAS, those who had been anxious in the past but were not currently anxious (*p* = 0.002) and those who identified as currently anxious (*p* ≤ 0.001) had higher levels of dental anxiety than respondents who had never experienced anxiety. Respondents who had personally experienced an unpleasant event, those who had indirectly acquired anxiety, those with an anxious personality (*p* ≤ 0.001) and those who had acquired anxiety in other ways (*p* = 0.008) had statistically significantly higher levels of dental anxiety than those without anxiety. No statistically significant difference in dental anxiety was found in relation to the requirement for analgesics, sedatives, or local anaesthesia before the procedure (*p* > 0.05).

Table 4 represents the self-assessment of dental health using the MDAS. Almost half of the participants (43.8%) rated their oral health as very good. The vast majority, 83.7% (n = 1298), consider oral health to be very important. Currently, 11.3% (n = 175) suffer from oral pain, while almost one-third of participants visit the dentist regularly (every 6 months). In terms of the MDAS, participants who reported excellent (*p* = 0.008) and very good (*p* = 0.024) oral health had statistically significantly lower anxiety scores than participants who reported poor oral health. In contrast, individuals who were currently experiencing some form of oral pain had significantly higher anxiety scores compared to those without pain (*p* ≤ 0.001). In addition, participants who went to the dentist irregularly had higher levels of anxiety than participants who went for regular check-ups (*p* ≤ 0.001).

**Figure 1:**
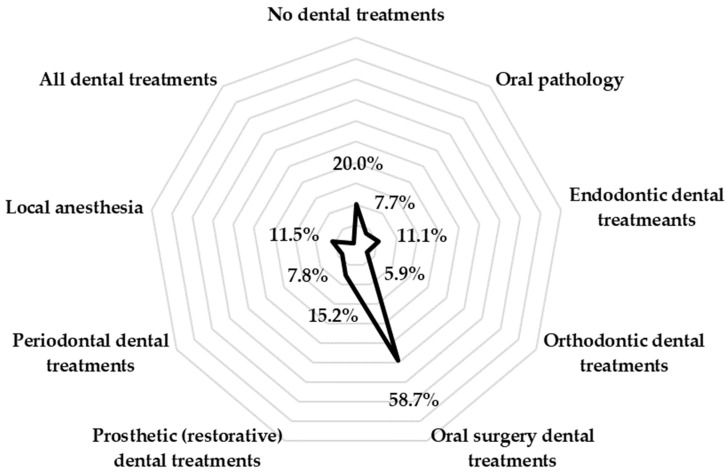
Most feared dental procedures.

Figure 1 shows which dental procedures patients fear the most, with multiple answers possible. More than half of the respondents (58.7%) stated that oral surgery causes them the greatest anxiety (n = 911). Conversely, a significantly lower percentage of respondents stated that orthodontic procedures (5.9% of them) and oral medicine procedures (7.7%) cause them the most anxiety.

**Figure 2:**
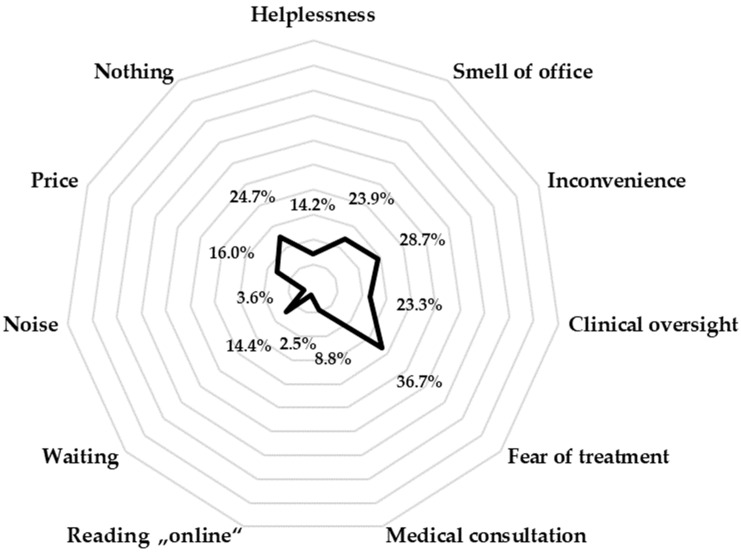
Dental-fear-related factors.

Figure 2 shows the factors that influence an increased fear of dentists, with the possibility of multiple answers. Fear of treatment is shown to be the most important factor related to fear of dentists (selected by 36.7 of respondents), while 28.7% of respondents consider discomfort to be the most important factor. In contrast, only 2.5% of respondents believe that online research has the greatest effect of increasing their fear of dental treatment.

Table 5 shows the participants’ subjective evaluation of their psychophysiological, behavioural, and emotional reactions during a dental visit. Almost all participants (92.8%) reported that they almost never faint in the dental office, while 25.7% of them reported that they occasionally have a dry mouth. Only 6.0% of participants reported that they are constantly unable to relax in the dental office.

Table 6 shows the avoidance of visits to the dentist due to fear. A significant percentage of participants, 40.6%, postpone dental visits for as long as possible. Just under one-fifth of the participants reported cancelling appointments due to anxiety. Interestingly, 15.3% of the respondents said that they had been persuaded by others to visit the dentist because they were in pain. Using the MDAS, it was found that participants who put off going to the dentist for as long as possible had a higher level of dental anxiety (*p* ≤ 0.001) than those who did not avoid going to the dentist. A statistically significant difference was observed in participants who stated that they avoided going to the dentist until they were in unbearable pain (*p* = 0.011).

## 4. Discussion

Considering that anxiety and fear of the dentist are widespread in all age groups and represent a significant barrier to maintaining appropriate oral hygiene habits and achieving favourable oral health outcomes [37], this study aimed to investigate the relationships between dental anxiety, self-perceived oral health status, and sociodemographic factors in the adult population in Croatia. According to the results of the MDAS, more than two-thirds of the participants belonged to the categories with no or low dental anxiety. The self-reported presence of dental anxiety proved to have a significant influence on the level of dental anxiety, while unpleasant experiences at the dentist and the time at which they occurred showed no significant influence. Participants who described their oral health as excellent or very good had lower levels of dental anxiety, while participants who went to the dentist irregularly showed higher levels of anxiety. It was also confirmed that participants with higher levels of dental anxiety tended to avoid dental visits for as long as possible or until they were in severe pain.

According to the MDAS, about one-fifth of the participants (19.1%) in this study belonged to the category without dental anxiety. The average score for dental anxiety according to the MDAS questionnaire was around 10 (median 8.0). Similar results were found in Finland [21], while slightly higher scores were found in Ireland [38], Lithuania, Norway [39], and Italy [40]. In a study conducted in Brazil [34], it was found that approximately 80.0% of participants had no dental anxiety at all. In this study, 7.8% of participants belonged to the group with severe dental anxiety or phobia, which is comparable to Poland [8] and the UK [41].

Although many previous studies have reported that dental anxiety is more common in women [8,9,34,42], the results of this study do not indicate a difference between the sexes. Dental anxiety in women is often associated with the presence of other phobias, higher levels of neuroticism, more severe psychiatric disorders, and higher levels of generalised anxiety. In addition, cultural factors may play an important role in the expression of anxiety, as it is considered more acceptable for women to express anxiety openly, whereas men are expected not to show pain or fear [9,43]. Similar results to this study were found in studies in the United Arab Emirates [4] and Bulgaria [14]. Age also plays a role in dental anxiety. Previous research has shown that younger people often have greater dental anxiety than older people, as older people tend to have more experience with dental treatment, which can reduce their anxiety [2,38]. However, this study finds the opposite, as does a study from the United Arab Emirates [4]. Moreover, it is common for people with a higher level of education to have less fear of the dentist because they understand the treatment procedures better than people with a lower level of education [1,6,15,38]. However, this study shows the opposite results. In particular, participants with higher academic degrees showed higher levels of anxiety, which was also observed in studies from the United Arab Emirates [4], Pakistan [16], the Netherlands [17], and India [7]. Numerous previous studies suggest that individuals from lower socioeconomic backgrounds have higher levels of anxiety, highlighting the influence of this factor on dental anxiety [9,10]. However, that was not confirmed in this study, nor in studies conducted in Sweden [18], Turkey [19], and Brazil [34].

The Modified Dental Anxiety Scale (MDAS) is used to assess participants’ level of anxiety using five questions [36]. More than half of the participants in this study stated that they would not feel anxious if they had a dental appointment tomorrow. In contrast, in two studies conducted on the adult population in India, a significantly lower percentage of participants said they were not anxious when asked the same question [10,44]. These studies found that just over 10.0% of participants felt very uncomfortable about having their teeth drilled. Similar results were confirmed in studies conducted on the adult population in the United Arab Emirates [4], Turkey [19], Denmark [20], and Italy [40].

Anxiety and fear related to dental procedures represent a complex clinical situation in which people with dental anxiety may have unexpectedly positive experiences, while people without anxiety may have traumatic experiences [40]. These findings make it necessary to identify patients with any form of fear of dental treatment in order to improve their safety and the quality of dental care. According to the results of this study, about one-quarter of the respondents said they were afraid of dental procedures, while less than one-fifth fell into groups with higher dental anxiety according to the MDAS. Just over 50.0% of the respondents reported unpleasant dental experiences, with a significant proportion of these experiences occurring for the first time in childhood and adolescence (approximately 65.0%). In Brazil [34] and Greece [45], a significantly lower number of respondents reported having had negative experiences in dental offices. The assertion that patients who have had negative experiences with the dentist in childhood and adolescence are more likely to develop anxiety compared to patients without such experiences is supported by studies conducted on adults in Lebanon [1], the Republic of Croatia [30], and the United Kingdom [38].

The self-reported presence of dental anxiety as well as the sources of this anxiety proved to have a significant influence on the level of dental anxiety among respondents, while the timing of the first unpleasant experience and the experience itself had no significant influence. In this study, 36.2% of the respondents had a personal unpleasant experience, while 11.2% had acquired their fear indirectly. A study conducted on the adult population of Poland [8], where almost half of the respondents had had an unpleasant experience, as well as studies in India [6] and China [46], came to similar conclusions. Personal unpleasant experiences appear to be the main route for the development of dental anxiety. Indirectly acquired fear represents an alternative pathway for fear acquisition in which the presence of a stimulus is not necessary. This mechanism could explain childhood dental anxiety, where children develop a fear of dental environments through exposure to family members with a dental phobia, by seeing negative portrayals of dental offices in various media, and from friends who report personal negative experiences [47]. This study has shown that indirectly acquired fear, reported by approximately 10.0% of the respondents, is associated with higher dental anxiety, which is consistent with the findings of studies from Spain [12] and the United States [13].

Participants in this study who rated their oral health as excellent or very good had lower levels of anxiety according to the MDAS, which is consistent with the results of other studies from the United Arab Emirates [4], Poland [8], and India [44]. However, the relationship between the quality of oral health and dental anxiety was not confirmed in a study conducted in China [46]. Pain has been shown to be a key factor contributing to the development of dental anxiety. In particular, the experience of pain prior to treatment can be associated with increased anxiety in patients [46]. Approximately 10.0% of respondents in our study reported currently experiencing pain in the oral cavity, and these respondents also exhibited higher levels of dental anxiety, which is consistent with the results of a study conducted in China. This study found that respondents who did not have regular dental check-ups had higher levels of dental anxiety. Similar data were collected in the United Arab Emirates [4], Turkey [9], the Netherlands [17], Sweden [18], and the United Kingdom [38]. Unplanned visits or emergencies can lead to increased anxiety due to the unexpected nature of the visit. On the other hand, patients with scheduled appointments may have more time to think about the procedure, which may increase their anxiety. However, the exact impact of a scheduled treatment on anxiety levels depends on various factors, such as the patient’s individual characteristics, previous experiences, and overall anxiety levels [4].

Various factors can play a role in the development of dental anxiety, with the exact causes varying from person to person. Patients treated in departments that specialise in non-invasive procedures (that do not require local anaesthesia or dental drilling) may be less likely to suffer from dental anxiety. Conversely, patients undergoing surgical and restorative procedures may find them uncomfortable and intimidating [48]. In our study, 58.7% of patients stated that oral surgery was the dental procedure that scared them the most, while just over one-tenth of the respondents identified local anaesthesia as the most frightening procedure. Similarities can be found with studies conducted in the United Arab Emirates [4], India [44], and Pakistan [49]. However, a study conducted on the adult population in India [6] showed that the type of dental procedure had no influence on the occurrence of major dental anxiety.

It is crucial to assess the patient’s ability to cope with uncomfortable dental situations, identify factors that contribute to anxiety, and evaluate their motivation to face dental anxiety. In this way, dentists can build a good relationship with the patient, determine appropriate strategies to manage dental anxiety, and consider the use of medication to alleviate the patient’s discomfort [37]. When asked what factors increased their fear of the dentist, more than one-third of respondents cited fear of the procedure as the main cause, followed by discomfort. In studies conducted on the adult population in Denmark [50], a significantly higher proportion of patients cited concern about possible injury, a feeling of helplessness during the dental procedure, and previous negative experiences with other dentists as factors contributing to dental phobia.

The different stages of dental anxiety require different approaches to patient care. Patients often report a feeling of choking when they have too many instruments in their mouth at once, or difficulty breathing when a rubber dam is in place. Some even describe a “reaction” or “allergy” to local anaesthetics, which can usually be interpreted as symptoms of autonomic nervous system arousal such as palpitations and shortness of breath [51]. More than 60.0% of the respondents in our study stated that they did not notice an increase in their breathing or pulse rate during the dental visit. A slightly higher percentage of respondents in Pakistan [48] and Saudi Arabia [52] reported the same.

The results of our study showed that 60.0% of the respondents do not avoid going to the dentist out of fear. Moreover, the vast majority do not miss or cancel a scheduled dental appointment due to fear, similar to in Pakistan [48]. In our study, respondents who reported avoiding dental visits had higher levels of dental anxiety, similar to those observed in Saudi Arabia [11] and Denmark [20].

This study has several limitations that need to be considered. First, the sample is limited to individuals who had internet access, which was required to complete the survey, potentially excluding older populations who may not have internet access or be active on social media. This restriction could have led to an underrepresentation of older adults in the sample. In addition, the survey took around 10 min to complete, which could have led to incomplete questionnaires or the survey being abandoned. In addition, the high proportions of women and younger adults in the sample may lead to bias and possible discrepancies in the results compared to a mixed-age and -gender population. The use of a closed questionnaire may also limit the depth and accuracy of the responses and may not provide a complete picture of the respondents’ attitudes. Furthermore, as the study was conducted via an online survey, there is a possibility that the respondents having self-reported led to subjective or biased responses. Finally, the lack of direct observation or interaction with the respondents meant we could not fully assess their actual attitudes and experiences.

Future research should investigate the specific causes and mechanisms underlying dental anxiety, to better understand its aetiology and development. In addition, more in-depth analyses of all socioeconomic variables that might influence the assessment of dental anxiety in the adult population should be conducted. Furthermore, future studies could investigate the roles of social support and patient education in reducing dental anxiety and develop personalised approaches that take into account individual patient characteristics. Integrating a multidisciplinary approach into future intervention programs could also help to improve the oral health and quality of life of people with dental anxiety.

## 5. Conclusions

The results of this study show that dental anxiety varies depending on demographic, socioeconomic, and oral health characteristics. One-fifth of the respondents reported no dental anxiety, while almost one-tenth were affected by dental phobia. The education level and employment status were found to be important factors influencing the level of anxiety. The greatest anxiety was triggered by dental drilling and oral surgery, with a dry mouth, inability to relax, and muscle tension among the most commonly reported symptoms. In addition, there is a significant correlation between high levels of anxiety and avoidance of dental visits due to severe pain. Recommendations for future research and practice include focusing on educating and informing patients about dental procedures, to reduce anxiety. In addition, developing strategies to identify and support patients with severe anxiety may improve their experience and oral health. Particular attention should be paid to patients who avoid dental visits due to anxiety, providing them with appropriate support and interventions to reduce their anxiety.

## Figures and Tables

**Table 1:** Demographic and socioeconomic characteristics of participants in relation to the level of dental anxiety and fear.

Characteristic		Frequency n (%)	MDAS Score β (95% CI)	*p*-Value
Gender	Male	365 (23.5)	Reference	
	Female	1186 (76.5)	0.201 (−0.038–0.440)	0.099
Age group (years)	18–30	688 (44.4)	Reference	
	31–50	577 (37.2)	−0.192 (−0.564–0.180)	0.312
	≥51	286 (18.4)	−0.164 (−0.461–0.132)	0.277
Education level	Elementary school	34 (2.2)	Reference	
	High school	740 (47.7)	0.528 (−0.177–1.233)	0.142
	Bachelor’s degree	234 (15.1)	0.942 (0.200–1.684)	0.013 *
	Master’s degree	473 (30.5)	0.472 (−0.244–1.189)	0.196
	MSc/PhD	70 (4.6)	0.914 (0.062–1.766)	0.035 *
Employment	Student	391 (25.2)	Reference	
	Unemployed	137 (8.8)	0.302 (−0.114–0.718)	0.155
	Employed	936 (60.3)	0.452 (0.123–0.782)	0.007 *
	Retired	87 (5.6)	0.362 (−0.234–0.958)	0.234
Socioeconomic status	Below average	70 (4.5)	Reference	
	Average	1289 (83.1)	−0.415 (−0.919–0.089)	0.107
	Above average	192 (12.4)	−0.473 (−1.046–0.100)	0.106

Data are presented as numbers (percentages) and as regression coefficients (95% confidence interval). The reference MDAS score category was “low”. MDAS, Modified Dental Anxiety Scale; β, regression coefficient. * Statistical significance was set at *p* < 0.05.

**Table 2:** Distribution of participants according to responses on the Modified Dental Anxiety Scale.

Question	Not Anxious n (%)	Slightly Anxious n (%)	Fairly Anxious n (%)	Very Anxious n (%)	Extremely Anxious n (%)
Treatment tomorrow	949 (61.2)	327 (21.1)	114 (7.4)	64 (4.1)	97 (6.3)
Waiting room	844 (54.4)	377 (24.3)	129 (8.3)	83 (5.4)	118 (7.6)
Tooth drilled	444 (28.6)	607 (39.1)	219 (14.1)	86 (5.5)	195 (12.6)
Teeth scaled and polished	848 (54.7)	387 (25.0)	148 (9.5)	77 (5.0)	91 (5.9)
Local anaesthetic injection	727 (46.9)	492 (31.7)	161 (10.4)	68 (4.4)	103 (6.6)

Q1: How would you feel if you knew you had a dental appointment tomorrow? Q2: How do you feel while sitting in the waiting room, waiting for your turn at the dentist? Q3: How do you feel during tooth drilling? Q4: How do you feel during tooth polishing? Q5: How would you feel if the dentist were to give you an aesthetic? Data are presented as numbers (percentages).

**Table 3:** Relationships between the presence of dental fear, negative dental experiences, and the level of dental anxiety.

Question (Characteristic)	Answer	Frequency n (%)	MDAS Score β (95% CI)	*p*-Value
Dental fear	Never	457 (29.5)	Reference	
	Used to be, not now	711 (45.8)	0.642 (0.241–1.043)	0.002 *
	Always	383 (24.7)	3.025 (2.390–3.659)	≤0.001 *
Aversive dental experience	No	740 (47.7)	Reference	
	Yes	811 (52.3)	0.093 (−0.210–0.395)	0.548
First aversive experience	Never	324 (20.9)	Reference	
	Childhood	820 (52.9)	0.301 (−0.211–0.814)	0.249
	Adolescence	198 (12.8)	0.422 (−0.189–1.033)	0.176
	Adult	102 (6.6)	0.190 (−0.515–0.895)	0.598
	Unknown	107 (6.9)	0.322 (−0.318–0.963)	0.324
Fear acquisition	No fear	546 (35.2)	Reference	
	Personal adverse experience	561 (36.2)	1.297 (0.909–1.684)	≤0.001 *
	Indirectly acquired	174 (11.2)	1.296 (0.842–1.750)	≤0.001 *
	Fearful personality	200 (12.9)	1.814 (1.313–2.316)	≤0.001 *
	Other	70 (4.5)	0.917 (0.243–1.591)	0.008 *
Pre-procedure analgesic use	Never	1243 (80.1)	Reference	
	Rarely	166 (10.7)	−0.008 (−0.432–0.416)	0.971
	Sometimes	107 (6.9)	0.627 (0.010–1.245)	0.051
	Often	14 (0.9)	20.458 (−35,576.386–35,617.303)	0.999
	Always	21 (1.4)	19.898 (−29,394.045–29,433.841)	0.999
Pre-procedure sedative use	Never	1440 (92.8)	Reference	
	Rarely	51 (3.3)	0.547 (−0.255–1.348)	0.181
	Sometimes	33 (2.1)	0.395 (−1.192–1.983)	0.626
	Often	10 (0.6)	−0.054 (−2.487–2.379)	0.965
	Always	17 (1.1)	19.786 (−29,629.003–29,668.575)	0.999
Pre-procedure local anaesthesia use	Never	420 (27.1)	Reference	
	Rarely	229 (14.8)	−0.174 (−0.582–0.235)	0.405
	Sometimes	301 (19.4)	0.071 (−0.303–0.445)	0.709
	Often	199 (12.8)	0.358 (−0.090–0.807)	0.117
	Always	402 (25.9)	0.058 (−0.318–0.435)	0.761

Data are presented as numbers (percentages) and regression coefficients (95% confidence interval). The reference MDAS score category was “low”. MDAS, Modified Dental Anxiety Scale; β, regression coefficient. * Statistical significance was set at *p* < 0.05.

**Table 4:** Self-assessment of dental association in relation to the level of dental anxiety and fear.

Question	Answer	Frequency n (%)	MDAS Score β (95% CI)	*p*-Values
Oral health	Excellent	235 (15.2)	−2.828 (−4.927–−0.729)	0.008 *
	Very good	679 (43.8)	−2.400 (−4.485–−0.315)	0.024 *
	Good	522 (33.7)	−1.740 (−3.820–0.339)	0.101
	Fair	99 (6.4)	−1.118 (−3.244–1.008)	0.303
	Poor	16 (1.0)	Reference	
Importance of oral health	Very important	1298 (83.7)	2.864 (−0.623–6.350)	0.107
	Moderately important	237 (15.3)	3.130 (−0.363–6.622)	0.079
	Somewhat important	14 (0.9)	24.302 (−39,546.848–39,595.452)	0.999
	Not important at all	2 (0.1)	Reference	
Number of extracted teeth	>10	74 (4.8)	−0.382 (−0.961–0.196)	0.195
	6–10	200 (12.9)	0.357 (−0.030–0.744)	0.071
	1–5	839 (54.1)	−0.016 (−0.267–0.234)	0.899
	0	438 (28.2)	Reference	
Number of teeth with fillings or crowns	>10	166 (10.7)	0.169 (−0.269–0.607)	0.450
	6–10	585 (37.7)	−0.094 (−0.420–0.232)	0.572
	1–5	560 (36.1)	0.146 (−0.178–0.471)	0.377
	0	240 (15.5)	Reference	
Gums bleeding	Yes	323 (20.8)	0.132 (−0.153–0.416)	0.365
	No	1228 (79.2)	Reference	
Bad breath	Yes	291 (18.8)	0.437 (0.135–0.738)	0.004 *
	No	1260 (81.2)	Reference	
Pain currently	Yes	175 (11.3)	0.953 (0.598–1.308)	≤0.001 *
	No	1376 (88.7)	Reference	
Frequency of dental check-ups	Regular	485 (31.3)	Reference	
	Irregular	1046 (67.4)	0.536 (0.305–0.767)	≤0.001 *
	Never	20 (1.3)	0.846 (−0.239–1.930)	0.126

Data are presented as numbers (percentages) and regression coefficients (95% confidence interval). The reference MDAS score category was “low”. MDAS, Modified Dental Anxiety Scale; β, regression coefficient. * Statistical significance was set at *p* < 0.05.

**Table 5:** Participants’ subjective assessment based on their psychophysiological, behavioural, and emotional reactions during dental visits.

Question	Almost Never n (%)	Sometimes n (%)	Often n (%)	Always n (%)
Wobbliness in legs	898 (57.9)	395 (25.5)	219 (14.1)	39 (2.5)
Black spots in field of vision	1339 (86.3)	115 (7.4)	86 (5.5)	11 (0.7)
Difficulty in breathing	1050 (67.7)	288 (18.6)	174 (11.2)	39 (2.5)
Feeling hot	1043 (67.2)	290 (18.7)	181 (11.7)	37 (2.4)
Nausea	1243 (80.1)	165 (10.6)	114 (7.4)	29 (1.9)
Heart pounding/racing	949 (61.2)	360 (23.2)	194 (12.5)	48 (3.1)
Dry mouth	834 (53.8)	399 (25.7)	260 (16.8)	58 (3.7)
Dizzy or lightheaded	1358 (87.6)	110 (7.1)	67 (4.3)	16 (1.0)
Indigestion	1099 (70.9)	270 (17.4)	137 (8.8)	45 (2.9)
Numbness or tingling	1315 (84.8)	138 (8.9)	78 (5.0)	20 (1.3)
Faint/lightheaded	1439 (92.8)	70 (4.5)	34 (2.2)	8 (0.5)
Unable to relax	920 (59.3)	320 (20.6)	218 (14.1)	93 (6.0)
Feeling of choking	1398 (90.1)	83 (5.4)	56 (3.6)	14 (0.9)
Hands trembling	1269 (81.8)	152 (9.8)	96 (6.2)	34 (2.2)
Face flushed	1209 (77.9)	196 (12.6)	117 (7.5)	29 (1.9)

Data are presented as numbers (percentages).

**Table 6:** Avoidance of dental visits in relation to the level of dental anxiety and fear.

Question	Answer	Frequency n (%)	MDAS Score β (95% CI)	*p*-Value
I avoided going to the dentist for as long as possible	Yes	630 (40.6)	1.673 (1.375–1.971)	≤0.001 *
	No	921 (59.4)	Reference	
I avoided going to the dentist until I felt excruciating pain	Yes	493 (31.8)	0.427 (0.099–0.755)	0.011 *
	No	1058 (68.2)	Reference	
I did not appear at the agreed appointment time	Yes	181 (11.7)	0.069 (−0.406–0.544)	0.776
	No	1370 (88.3)	Reference	
I cancelled the appointment	Yes	250 (16.1)	0.065 (−0.328–0.458)	0.746
	No	1301 (83.9)	Reference	
I arrived at the dental office but left before entering	Yes	98 (6.3)	−0.232 (−0.814–0.349)	0.434
	No	1453 (93.7)	Reference	
Finally, someone else convinced me to visit the dentist because I was in pain	Yes	238 (15.3)	0.368 (−0.062–0.799)	0.094
	No	1313 (84.7)	Reference	

Data are presented as numbers (percentages) and regression coefficients (95% confidence interval). The reference MDAS score category was “low”. MDAS, Modified Dental Anxiety Scale; β, regression coefficient. * Statistical significance was set at *p* < 0.05.

## Data Availability

Data are available upon request from the corresponding author.

## References

[1] Kassem El Hajj H., Fares Y., Abou-Abbas L. (2021). Assessment of dental anxiety and dental phobia among adults in Lebanon. BMC Oral Health.

[2] Silveira E.R., Cademartori M.G., Schuch H.S., Armfield J.A., Demarco F.F. (2021). Estimated prevalence of dental fear in adults: A systematic review and meta-analysis. J. Dent..

[3] Sun I.G., Chu C.H., Lo E.C.M., Duangthip D. (2024). Global prevalence of early childhood dental fear and anxiety: A systematic review and meta-analysis. J. Dent..

[4] Alansaari A.B.O., Tawfik A., Jaber M.A., Khamis A.H., Elameen E.M. (2023). Prevalence and socio-demographic correlates of dental anxiety among a group of adult patients attending dental outpatient Clinics: A Study from UAE. Int. J. Environ. Res. Public Health.

[5] Randall C.L., Shaffer J.R., McNeil D.W., Crout R.J., Weyant R.J., Marazita M.L. (2017). Toward a genetic understanding of dental fear: Evidence of heritability. Community Dent. Oral Epidemiol..

[6] Malvania E.A., Ajithkrishnan C.G. (2011). Prevalence and socio-demographic correlates of dental anxiety among a group of adult patients attending a dental institution in Vadodara city, Gujarat, India. Indian J. Dent. Res..

[7] Saheer A., Majid S.A., Raajendran J., Chithra P., Chandran T., Mathew R.A. (2022). Effect of dental anxiety on oral health among the first-time dental visitors: A hospital-based study. J. Pharm. Bioallied Sci..

[8] Sopińska K., Bołtacz-Rzepkowska E. (2016). Dental fear and its effect on health behaviour of adult patients in Lodz region. J. Stomatol..

[9] Yildirim T.T. (2016). Evaluating the relationship of dental fear with dental health status and awareness. J. Clin. Diagn. Res..

[10] Shrienitha D.N., Sudhir K.M., Prasad S.V., Sreenidhi S., Mahesh J., Priyadharshini K.I. (2021). Assessment of dental anxiety level among adult patients visiting Dental College in Chengalpet District-a questionnaire survey. Oral Health Dent. Manag..

[11] Alenezi A.A., Aldokhayel H.S. (2022). The impact of dental fear on the dental attendance behaviors: A retrospective study. J. Fam. Med. Prim. Care.

[12] Lara A., Crego A., Romero-Maroto M. (2012). Emotional contagion of dental fear to children: The fathers’ mediating role in parental transfer of fear. Int. J. Paediatr. Dent..

[13] McNeil D.W., Randall C.L., Cohen L.L., Crout R.J., Weyant R.J., Neiswanger K., Marazita M.L. (2019). Transmission of dental fear from parent to adolescent in an Appalachian sample in the USA. Int. J. Paediatr. Dent..

[14] Kirova D.G., Atanasov D.T., Lalabonova C.K., Janevska S. (2010). Dental anxiety in adults in Bulgaria. Folia Med..

[15] Deogade S., Suresan V. (2016). Psychometric assessment of anxiety with the Modified Dental Anxiety scale among central Indian adults seeking oral health care to a dental school. Ind. Psychiatry J..

[16] Muneer M.U., Ismail F., Munir N., Shakoor A., Das G., Ahmed A.R., Ahmed M.A. (2022). Dental anxiety and influencing factors in adults. Healthcare.

[17] Stouthard M.E.A., Hoogstraten J. (1990). Prevalence of dental anxiety in the Netherlands. Community Dent. Oral Epidemiol..

[18] Hakeberg M., Berggren U., Carlsson S.G. (1992). Prevalence of dental anxiety in an adult population in a major urban area in Sweden. Community Dent. Oral Epidemiol..

[19] Tunc E.P., Firat D., Onur O.D., Sar V. (2005). Reliability and validity of the Modified Dental Anxiety Scale (MDAS) in a Turkish population. Community Dent. Oral Epidemiol..

[20] Moore R., Birn H., Kirkegaard E., Brødsgaard I., Scheutz F. (1993). Prevalance and characteristics of dental anxiety in Danish adults. Community Dent. Oral Epidemiol..

[21] Somero A., Suominen A., Pohjola V., Ogawa M., Sipilä K., Kakko N., Tulppo M., Lahti S. (2024). Autonomic nervous system activity and dental anxiety in the Northern Finland birth cohort (NFBC1966) population. Dent. J..

[22] Chi S.I. (2023). What is the gold standard of the dental anxiety scale?. J. Dent. Anesth. Pain Med..

[23] Šimunović L., Špiljak B., Radulović M., Vlahovljak A., Ostojić M., Krlev J., Ibrahimpašić A., Vranić L., Negovetić Vranić D. (2022). Relationship between children’s and parents’ dental anxiety: A cross-sectional study on the six European countries. Dent. J..

[24] Petrović D., Cicvarić O., Šimunović-Erpušina M., Ivančić Jokić N., Bakarčić D., Bučević Sojčić P., Jurić H. (2024). The role of family factors in the development of dental anxiety in children. Medicina.

[25] Škrinjarić T., Leko J., Goršeta K. (2019). Dental anxiety in children in relation to dental health. Coll. Antropol..

[26] Majstorovic M., Veerkamp J.S.J., Skrinjaric I. (2003). Reliability and validity of measures used in assessing dental anxiety in 5- to 15-year-old Croatian children. Eur. J. Paediatr. Dent..

[27] Leko J., Škrinjarić T., Goršeta K. (2020). Reliability and validity of scales for assessing child dental fear and anxiety. Acta Stomatol. Croat..

[28] Katanec T., Singh S., Majstorovic M., Klaric I., Herman N.G., Moursi A.M. (2018). Gender differences in dental anxiety and medical fear in Croatian adolescents. J. Clin. Pediatr. Dent..

[29] Majstorović M., Skrinjarić T., Szirovicza L., Glavina D., Veerkamp J.S. (2007). Dental anxiety in relation to emotional and behavioral problems in Croatian adolescents. Coll. Antropol..

[30] Surina K., Ruždijić J., Kuzinovska A., Stevanović A., Rončević-Gržeta I. (2021). Fear of going to the dentist. Soc. Psihijat..

[31] Eysenbach G. (2004). Improving the quality of Web surveys: The Checklist for Reporting Results of Internet E-Surveys (CHERRIES). J. Med. Internet Res..

[32] State Statistical Office Estimates of the Population of the Republic of Croatia in 2022. https://podaci.dzs.hr/media/25hinamc/stan-2023-3-1-procjena-stanovni%C5%A1tva-republike-hrvatske-u-2022.pdf.

[33] Dempster L.J. (2007). Measurement and Characterization of Fear and Avoidance in Dental Anxiety. Ph.D. Thesis.

[34] Penteado L.A.M., Pinho R.C.M., Dos Santos N.B., Vajgel B.C.F., Cimões R. (2018). The impact of dental anxiety and dental fear on the periodontal status and quality of life among dental patients. Braz. J. Oral Sci..

[35] Beck A.T., Epstein N., Brown G., Steer R.A. (1988). An inventory for measuring clinical anxiety: Psychometric properties. J. Consult. Clin. Psychol..

[36] Sivaramakrishnan G., Makki H., AlDallal S., Alaswad Z., Sultan E., Ahmed S., AlBanna H., Alsobaiei M., AlSalihi L. (2022). The variables associated with dental anxiety and their management in primary care dental clinics in Bahrain: A cross-sectional study. BMC Oral Health.

[37] Murad M.H., Ingle N.A., Assery M.K. (2020). Evaluating factors associated with fear and anxiety to dental treatment—A systematic review. J. Fam. Med. Prim. Care.

[38] Humphris G.M., Freeman R., Campbell J., Tuutti H., D’Souza V. (2000). Further evidence for the reliability and validity of the Modified Dental Anxiety Scale. Int. Dent. J..

[39] Stangvaltaite-Mouhat L., Stankeviciene I., Martinussen S.S.S., Sabataitis V., Sandjord C., Toresen I., Tryggestad M.S., Puriene A., Johnsen J.K. (2023). Web-based interventions reduced dental anxiety among adults in Lithuania and Norway: A pilot study. Int. J. Environ. Res. Public Health.

[40] Facco E., Gumirato E., Humphris G., Stellini E., Bacci C., Sivolella S., Cavallin F., Zanette G. (2015). Modified Dental Anxiety Scale: Validation of the Italian version. Minerva Stomatol..

[41] Heidari E., Banerjee A., Newton J.T. (2015). Oral health status of non-phobic and dentally phobic individuals; a secondary analysis of the 2009 Adult Dental Health Survey. Br. Dent. J..

[42] Saatchi M., Abtahi M., Mohammadi G., Mirdamadi M., Binandeh E.S. (2015). The prevalence of dental anxiety and fear in patients referred to Isfahan Dental School, Iran. Dent. Res. J..

[43] Hägglin C., Hakeberg M., Hällström T., Berggren U., Larsson L., Waern M., Pálsson S., Skoog I. (2001). Dental anxiety in relation to mental health and personality factors. A longitudinal study of middle-aged and elderly women. Eur. J. Oral Sci..

[44] Shitole S., Mounesh D., Veerabhadrappa S., Parkar M., Pankaj S.R. (2015). Assessment of dental anxiety in patients undergoing surgical extraction of teeth: Study from Western Maharashtra. Br. Biomed. Bull..

[45] Makri C., Alexias G., Togas C., Chasiotis V. (2020). Evaluation of dental anxiety and of its determinants in a Greek sample. Int. J. Caring Sci..

[46] Dou L., Vanschaayk M.M., Zhang Y., Fu X., Ji P., Yang D. (2018). The prevalence of dental anxiety and its association with pain and other variables among adult patients with irreversible pulpitis. BMC Oral Health.

[47] Carter A.E., Carter G., Boschen M., AlShwaimi E., George R. (2014). Pathways of fear and anxiety in dentistry: A review. World J. Clin. Cases.

[48] Taqi M., Zaidi S.J.A., Javaid J., Alam Z., Saleem A., Khan S.A. (2023). Patient perceptions and experiences of dental fear of different dental specialties: A mixed-method study. BMC Oral Health.

[49] Ali S., Farooq I., Khan S.Q., Moheet I.A., Al-Jandan B.A., Al-Khalifa K.S. (2015). Self-reported anxiety of dental procedures among dental students and its relation to gender and level of education. J. Taibah Univ. Med. Sci..

[50] Moore R., Brødsgaard I. (1997). Adult dental anxiety and related dentist beliefs in Danish private practices. Tandlaegebladet.

[51] Armfield J.M., Heaton L.J. (2013). Management of fear and anxiety in the dental clinic: A review. Aust. Dent. J..

[52] Hakeem F.F., Bhayat A., Al Shaar M.B.A., Qobaly A. (2016). Self-assessment of dental anxiety and fear among dental students in a Saudi Arabian College. J. Adv. Med. Med. Res..

